# Gα_i_ Signaling Promotes Marginal Zone B Cell Development by Enabling Transitional B Cell ADAM10 Expression

**DOI:** 10.3389/fimmu.2018.00687

**Published:** 2018-04-11

**Authors:** Il-Young Hwang, Cedric Boularan, Kathleen Harrison, John H. Kehrl

**Affiliations:** ^1^B-Cell Molecular Immunology Section, Laboratory of Immunoregulation, National Institutes of Allergy and Infectious Diseases, National Institutes of Health, Bethesda, MD, United States; ^2^InvivoGen, Toulouse, France

**Keywords:** heterotrimeric G-protein, Notch2, ADAM10, B lymphocyte, marginal zone

## Abstract

The follicular (FO) versus marginal zone (MZ) B cell fate decision in the spleen depends upon BCR, BAFF, and Notch2 signaling. Whether or how G_i_ signaling affects this fate decision is unknown. Here, we show that direct contact with Notch ligand expressing stromal cells (OP9-Delta-like 1) cannot promote normal MZ B cell development when progenitor B cells lack Gα_i_ proteins, or if Gi signaling is disabled. Consistent with faulty ADAM10-dependent Notch2 processing, Gα_i_-deficient transitional B cells had low ADAM10 membrane expression levels and reduced Notch2 target gene expression. Immunoblotting Gα_i_-deficient B cell lysates revealed a reduction in mature, processed ADAM10. Suggesting that Gα_i_ signaling promotes ADAM10 membrane expression, stimulating normal transitional B cells with CXCL12 raised it, while inhibiting Gα_i_ nucleotide exchange blocked its upregulation. Surprisingly, inhibiting Gα_i_ nucleotide exchange in transitional B cells also impaired the upregulation of ADAM10 that occurs following antigen receptor crosslinking. These results indicate that Gα_i_ signaling supports ADAM10 maturation and activity in transitional B cells, and ultimately Notch2 signaling to promote MZ B cell development.

## Introduction

The fetal liver and the bone marrow support B1 and B2 lymphocyte development (1, 2). Fetal liver hematopoietic progenitors give rise to long-lived B1 B lymphocytes, which predominately reside in serosal lined cavities and in the spleen. As hematopoiesis shifts from the fetal liver to the bone marrow, B1 B cell production largely ceases, and bone marrow progenitors produce B2 cells, which have two developmental fates that are not realized until the newly formed B cells traffic to the spleen. These recent splenic arrivals are termed transitional B cells and they express B220, CD19, IgM, and CD93 (3). They must undergo a second tolerance checkpoint and will eventually mature to a follicular (FO) or a marginal zone (MZ) B cell (4). FO B cells reside in lymphoid follicles and continuously recirculate between lymphoid organs. MZ B cells do not recirculate but can shuttle between the splenic MZ and follicles (5). They have a limited antigen receptor repertoire, largely specialized to recognize encapsulated bacteria and polysaccharide antigens in a T cell-independent fashion (6).

Precisely when the B2 cell developmental fate is assigned in the spleen has been controversial (7–9). Bone marrow-derived B cell progenitors enter the marginal sinus and red pulp of the spleen and are termed transitional B cell type 0 (T0) cells (10). T0 cells migrate from the MZ sinus into the splenic white pulp, where they acquire surface IgD becoming T1 cells. T1 cells that gain CD23 are termed transitional B cell type 2 (T2) cells and their survival is highly BAFF dependent (11). Based on high or low expression of CD21, T2 cells are divided into T2-MZ and T2-FO, respectively (3). As designated, these cells are biased to become FO or MZ B cells; however, the commitment to a MZ or FO B cell phenotype likely occurs before the T2 stage. A recent study exploring the loss of MZ B cells in thousand-and-one amino acid kinase (TAOK) 3-deficient mice found that wild-type T1 cells bearing surface ADAM10 exclusively differentiated into MZ B cells, while ADAM10 surface-negative T1 cells gave rise to both FO and MZ B cells (12).

A variety of genetic mutations bias B2 B cell differentiation toward either a MZ or FO B cell fate (13–18). These mutations largely impact Notch2, BCR, or BAFF signaling. A MZ fate requires the engagement of Notch2 on transitional B cells by Delta-like 1 (DL1). Splenic fibroreticular cells located in the white pulp express DL1 and their genetic ablation blocks MZ B cell development (19). Ligand binding to Notch2 induces an ADAM10-dependent cleavage that allows a subsequent intramembranous cleavage by γ-secretase. ADAM10^−/−^ mice lack MZ B cells (14, 20). The ADAM10 and γ-secretase-mediated cleavages releases the intracellular domain of Notch2 (NICD2), which enters the nucleus to interact with DNA-binding proteins to activate Notch2 target genes. Notch2 along with nuclear factor-κB (NF-κB) activation downstream of the BAFF receptors instructs a MZ fate (21). BCR signaling during positive selection may also impact the B2 cell fate decision as weak signals promote MZ B cell development while stronger signals bias the cells toward a FO B cell, perhaps by tuning the sensitivity of the cells to Notch2 signaling (15, 22). The loss of Gα_i_ proteins in developing B cells severely reduces MZ B cell numbers; however, whether or how this loss affects the MZ versus FO B cell fate decision is unknown (23, 24).

In B cells, the absence of Gα_i2_ markedly reduces the available pool of Gi proteins, and the additional loss of Gα_i3_ depletes it, disabling chemoattractant receptor signaling (24). Surprisingly, the absence of the three highly expressed B cell chemoattractant receptors CXCR5, CCR7, and EBI2, whose combined loss completely disrupts the splenic lymphoid architecture, did not cause a loss of phenotypic MZ B cells (25). Two G protein-coupled receptors (GPCRs) have been linked to MZ B cell positioning and retention, the cannabinoid receptor CB2 and the sphingosine 1-phosphate receptor 1 (S1PR1). Genetic targeting CB2 reduced MZ B cell numbers, while the loss of S1PR1 had no impact (5, 26–28). Despite the reduction in MZ B cells, the loss of CB2 did not affect BCR or Notch2 signaling (26). Targeting another GPCR, GPR97, caused an expansion of MZ B cells at the expense FO B cells, perhaps by affecting NF-κB activity (29). Together, the GPCR gene targeting data suggest that the loss of Gα_i_ in B cells causes the loss of MZ B cells due to their failure to be retained in the MZ, rather than impacting MZ B cell development.

In this study, we tested whether the loss of Gα_i_ proteins directly affected MZ B cell development. We show that despite the direct exposure to Notch2 ligand expressing cells, neither Gα_i_-deficient nor pertussis toxin (PTX)-treated progenitor B cells properly differentiate to MZ B cells. Favoring an intrinsic defect in Notch2 signaling, the Gα_i_-deficient transitional B cells have low levels of surface ADAM10 and of Notch2 target genes. Furthermore, treating B cell progenitors with PTX causes a partial T1–T2 transition block and inhibited the normal increase in ADAM10 that occurs on transitional B cells. The significance of these results is discussed.

## Materials and Methods

### Animals

C57BL/6J and C57BL/6J *vav1-cr*e mice were obtained from Jackson Laboratory. Mice with targeted deletion in *Gnai3^−/−^* and *Gnai2^−/−^*, and *Gnai2*^fl/fl^ mice were kindly provided by Dr. Lutz Birnbaumer (NIEHS, NIH) and backcrossed more than 12 times to C57BL/6J mice. *Gnai2*^fl/fl^*mb1-cre* and *Gnai2*^fl/fl^*vav1-cre* mice were obtained by crossing the appropriate cre expressing strain with the *Gnai2^fl/fl^* mice and backcrossing to obtain the desired genotype. *ric8*^fl/fl^ mice on a mixed background have been described previously and were backcrossed 10 times on to C57BL/6J. The C57BL/6 *mb1-cre* mice were kindly provided by Dr. Michael Reth (University of Freiburg, Germany). For those experiments that directly compared WT and gene targeted mice, littermate controls were used when possible. Otherwise age and sex matched *mb1-cre, vav1-cre, ric8^fl/fl^*, or *Gnai2^fl/fl^* mice served as controls. All mice were used in this study were 6–14 weeks of age. Mice were housed under specific-pathogen-free conditions. All the animal experiments and protocols used in the study were approved by the NIAID Animal Care and Use Committee at the National Institutes of Health.

### Cells and Cell Culture

OP9 control and OP9-DL1 cells were obtained from Dr. Juan-Carlos Zúñiga-Pflücker (University of Toronto) and maintained in α-MEM containing 20% fetal calf serum (FCS), antibiotics (100 IU/ml penicillin and 100 µg/ml streptomycin), 1 mM sodium pyruvate, and 50 µM 2-mercaptoethanol. Splenic B cells were isolated by negative depletion using biotinylated antibodies to CD4, CD8, CD11b, and CD11c and Dynabeads M-280 Streptavidin (Thermo Fisher Scientific). The B cell purity was greater than 95%. When needed B cells were cultured in RPMI 1640 containing 10% FCS (Gibco), 2 mM l-glutamine, antibiotics (100 IU/ml penicillin and 100 µg/ml streptomycin), 1 mM sodium pyruvate, and 50 µM 2-mercaptoethanol. Bone marrow cells were cultured in complete Iscove’s Modified Dulbecco’s Medium (GIBCO) in the presence of IL-7 (20 ng/ml) for 4 days to enrich for IgM^+^ immature B cells. On occasion, PTX (200 ng/ml) was added for the terminal 2 or 24 h of the culture. Subsequently, cells were washed twice with PBS and plated at 1 × 10^6^ cells/ml with 20 ng/ml of recombinant mouse BAFF (R&D Systems) in co-culture with OP9 or OP9-DL1 cells. The following were added, or not, to the cultures: Gallein (various concentrations, Tocris Bioscience), IBMX (1 µM, 3-Isobutyl-1-methylxanthine, Sigma-Aldrich), terbutaline (10 nM, Sigma-Aldrich), AM630 (1 µM, Tocris Bioscience), AMD3100 (1 µg/ml, Sigma-Aldrich), and CXCL12 (1 µg/ml, R&D Systems). The chemical compounds and BAFF were re-added on alternative days for the duration of the culture.

### Flow Cytometry and Antibodies

Single cells were resuspended in PBS, 2% FBS, and stained with fluorochrome-conjugated or biotinylated antibodies against B220 (RA3-6B2), IgD (11-26c-2a), IgM (R6-60.2), CD1d (K253), CD24 (M1/69), CD4 (GK1.5), CD8 (53-6.7), CD11c (HL3), CD11b (M1/70), CD19 (1D3), Notch-2 (16F11), CD93 (AA4.1), BP-1 (6C3), CD21/35 (4E3), CD23 (B3B4), CD43 (S7), and ADAM10 (all from Biolegend, BD Pharmingen, Thermo Fisher Scientific or R&D Systems). Biotin-labeled antibodies were visualized with fluorochrome-conjugated streptavidin (Thermo Fisher Scientific). LIVE/DEAD^®^ Fixable Aqua Dead Cell Stain Kit (Thermo Fisher Scientific) was used in all experiments to exclude dead cells. Compensation was performed using CompBeads (BD Biosciences) and ArC™ Amine Reactive Compensation Bead individually stained with each fluorochrome. Compensation matrices were calculated with FACSdiva software. Data acquisitions were done on FACSCanto II (BD) flow cytometer and analyzed with FlowJo software version 9 (Treestar).

### Intracellular Flow Cytometry

Labeling of dead cells, fixation, and permeabilization were performed as described in the manufacturer’s protocol. For the ADAM10 upregulation and detection of the level of phosphorylated signaling molecules, total splenocytes or purified B cells were rested in DMEM containing 1% FCS antibiotics (100 IU/ml penicillin and 100 µg/ml streptomycin), 1 mM sodium pyruvate, and 50 µM 2-mercaptoethanol for 30 min at 37°C/5% CO_2_ before stimulation with 1 µg/ml CXCL12 (R&D Systems) or α-IgM [F(ab′)_2_ Fragment goat Anti-mouse IgM, μ chain specific; Jackson ImmunoResearch]. Depending upon the experiment, cells were surface stained with anti-B220, anti-CD19, anti-CD21, anti-CD23, IgD, IgM, and anti-CD93 for 30 min at 4°C and following permeabilization, with anti-pTaok1-3 (EPR4883; Abcam), anti-pBTK-PE (N35-86; BD Biosciences), anti-pCREB-AlexaFluor 488 (J151-21; BD Biosciences), anti-pSyk-PE (moch1ct; Thermo Fisher Scientific), or pAkt Alexa Fluor 647 (D9E; Cell Signaling Technology) for 30 min at room temperature. For detection of pTAOK1-3, we used secondary F(ab′)_2_ fragment of goat anti-Rabbit IgG(H + L) (Thermo Fisher Scientific). Cells were finally resuspended in 250-µl 1% BSA/PBS and filtered prior to acquisition on a FACS Canto II flow cytometer (BD Biosciences).

### RNA Isolation and Real-Time qPCR

Splenic B-cell subsets were sorted, and RNA was isolated using the TRIzol (Thermo Fisher Scientific) according to the manufacturer’s instructions. Complementary DNA was synthesized using oligo(dT) and Omniscript RT (Qiagen) from 500 ng or 1 µg of total RNA. The real-time PCR primers used to amplify genes are listed below while those for *Dtx1, Id2, Id3, Bcl3, Btk, Hes1*, and *Rpl27* were from Qiagen (QuantiTect primer QT00097139, QT01038870, QT00248185, QT00247583, QT00102179, QT00313537, and QT02328683, respectively). Real-time PCR was performed using a QuantStudio 3 Real-Time PCR System (Thermo Fisher Scientific) following the Rotor-Gene SYBR Green PCR kit (Qiagen) protocol. All results are expressed as 2^−ΔΔCt^, where ΔΔCt = (Ct_target_ − Ct_Rpl27_) for treated samples—(Ct_target_ − Ct_Rpl27_) for control cells. Ct stands for cycle threshold. Data are shown as the mean ± SEM.

### Immunoblotting

The cells were lysed in RIPA buffer (Sigma-Aldrich) with 1 mM NaF, 1 mM PMSF, 1 mM DTT, and 1 mM Na_3_VO_4_ with complete and PhosSTOP inhibitors (Sigma-Aldrich). The lysates were separated by SDS-PAGE and transferred to nitrocellulose membranes by the iBLOT gel transfer system (Thermo Fisher Scientific). The membrane was incubated with 5% non-fat milk (w/v) in TBS buffer [25 mM Tris–HCl (pH 7.5), 150 mM NaCl, 0.1% Tween 20] for 1 h and then reacted with the primary Ab in TBS buffer with 2.5% non-fat milk or 5% BSA (w/v) for overnight by shaking at 4°C. The appropriated second Abs conjugated to HRP were used to detect the protein of interest *via* ECL. The following antibodies were used: ADAM10 (EPR5622; Abcam), Notch2 (C651.6DbHN-c; Developmental Studies Hybridoma Bank), pTAOK1-3 (EPR48883; Abcam), anti-actin-HRP conjugate (Sigma-Aldrich), Goat anti-rabbit IgG (H + L)-HRP conjugate #7074, 1:3,000 (Cell Signaling); and horse anti-mouse IgG, HRP-linked antibody #7076, 1:3,000 (Cell Signaling).

### Chemotaxis Assays

Chemotaxis assays were performed using a transwell chamber (Costar). Briefly, splenic B cells were immunostained for B cell subsets with fluorochrome-conjugated antibodies against B220, CD21, and CD23; washed twice, resuspended in complete RPMI 1640 medium, and added in a volume of 100 µl to the upper wells of a 24-well transwell plate with a 5-µm insert. Lower wells contained various concentrations of CXCL12 or CXCL13 (R&D Systems) in 600 µl of complete RPMI 1640 medium. The numbers of cells that migrated to the lower well after 2 h incubation were counted using a MACSQuant flow cytometer (Miltenyi Biotec). The percent migration was calculated as the numbers of cells that migrated into the lower part of the chamber divided by the total number of cells in the starting cell suspension, and multiplying the results by 100.

### ImageStream (IS): Labeling, Acquisition, and Analysis

The splenic B cells were stained with B220 PE conjugated, CD21 e450 conjugated and CD23 PE-cy7 conjugated for 10 min then fixed using 2% paraformaldehyde. The p65 subunit of NF-κB was visualized by indirect labeling. The primary Rabbit polyclonal NF-κB/p65 antibody (SantaCruz Biotechnology) was diluted 1:50 in 3% BSA, and 0.1% Triton X-100 in PBS and added to 1 × 10^6^ fixed cells. Samples were incubated for 2 h at room temperature. Primary antibody was washed and 1:200 dilution of secondary Alexa488-conjugated donkey anti rabbit IgG antibody (Jackson ImmunoResearch Laboratories Inc.) was added and incubated at room temperature in the dark for 1 h. Secondary antibody was removed, and cells resuspended in 100 µl PBS. Just prior to running on the IS, all samples had Draq5 (Cell Signaling) added (20 nM final concentration), to visualize the nucleus. At least, 20,000 events were collected for all samples on an IS MarkII using 488, 405, and 642 nm laser excitations. Cell populations were hierarchically gated for single cells that were in focus and were positive for both Draq5 and p65. Then, based on Draq5 intensity histogram, cells were gated on those that were in G0 and G1/S phase to avoid cells that have a disrupted nuclear membrane. After those gates were applied at least 1,000 cells per group were acquired (follicular: B220^+^CD23^+^CD21^−^, transitional: B220^+^CD23^−^CD21^−^, and MZ: B220^+^CD23^−^CD21^+^). Following data acquisition, the spatial relationship between the NF-κB and nuclear images was measured using a feature (ratio translocation) calculated in the IDEAS software package. The “Ratio translocation” feature, is the ratio of NF-κB intensity in the nuclear mask over NF-κB intensity in the whole cells (calculated using the brightfield mask) expressed as the percentage. Nuclear mask was defined based on Draq5 intensity where a signal was considered specific when it was a least 20 times higher than the background. The shift in the distribution between two populations (e.g., control versus treated cells) was tested using the Fisher’s Discriminant ratio calculated using Graphpad Prism software. A cell was considered with a nuclear p65 NF-κB when the “Ratio translocation” was greater than 60%.

### Statistics

*In vivo* results represent samples from three to nine mice per experimental group. Results represent mean values of at least triplicate samples. SEMs and *p* values were calculated with *t*-test or two-way ANOVA using GraphPad Prism (GraphPad software).

## Results

### Models of Gα_i_ Deficiency and Loss of MZ B Cells

**Figure 1:**
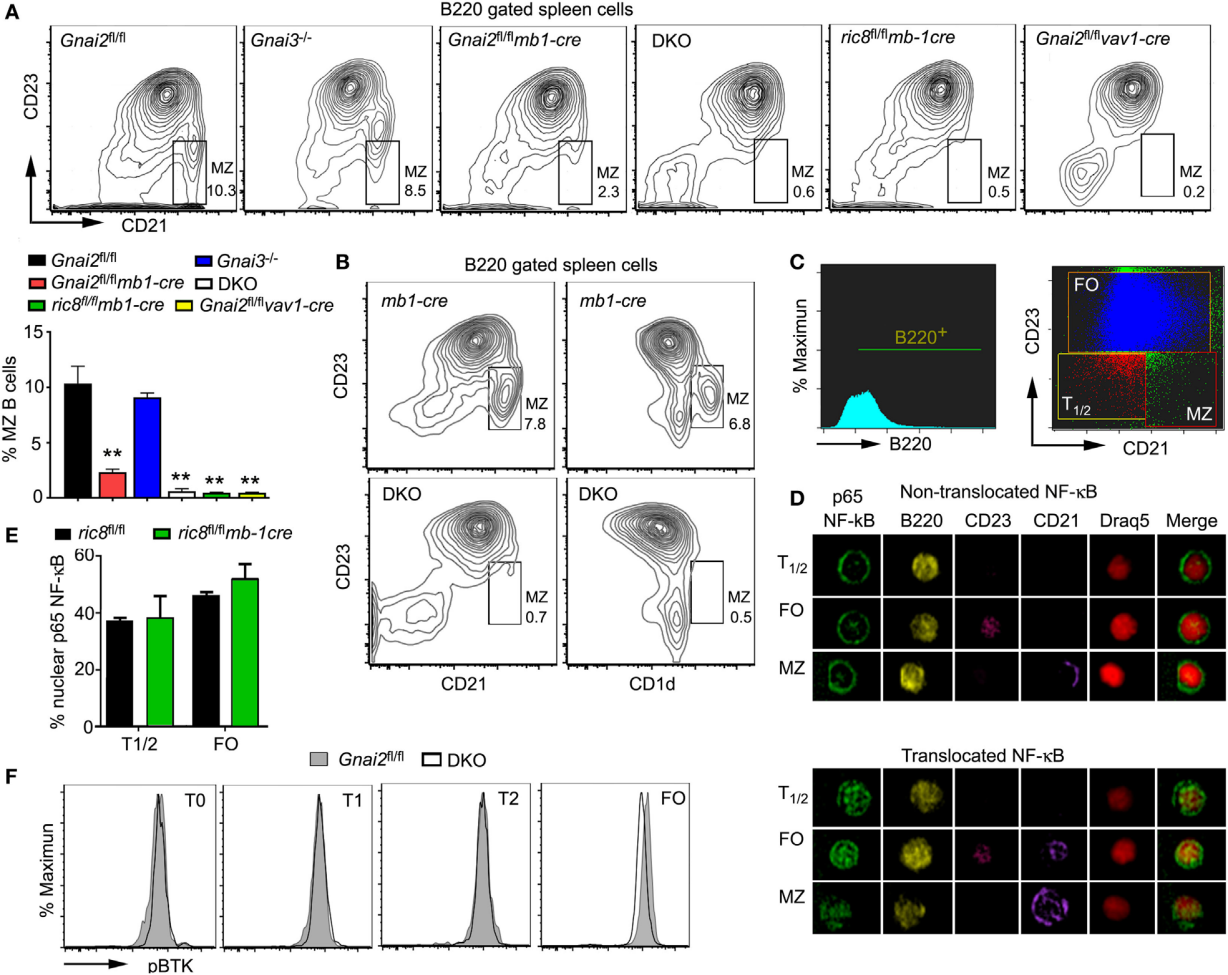
Despite the reduction in splenic marginal zone (MZ) B cells in Gα_i_-deficient mice, their transitional B cells do not exhibit evidence of reduced BCR signaling. **(A)** Lack of MZ B cells in various models of Gα_i_ deficiency. Flow cytometry patterns using splenic B cells from Gα_i_-deficient mice. CD23 versus CD21 on WT (Gnai2^fl/fl^), *Gnai3^−/−^, Gnai2*^fl/fl^*mb1-cre*, double knockout (DKO), *ric8*^fl/fl^*mb1-cre*, and *Gnai2*^fl/fl^*vav1-cre* B220^+^ splenocytes. The normal location of MZ B cell is boxed and % of cells indicated. The chart shows percentage of MZ B cells from the B220^+^ cell gate. Results are from nine WT, five *Gnai3^−/−^*, five *r*ic8^fl/fl^*mb1-cre*, eight *Gnai2*^fl/fl^*mb1-cre*, four *Gnai2*^fl/fl^*vav1-cre*, and four DKO mice. Data shown as mean ± SEM (**p < 0.005). **(B)** Spleens from Gα_i_-deficient mice also lack CD1d-positive cells. Flow cytometry patterns using splenic B cells from WT and DKO mice. CD23 versus CD21 and CD23 versus CD1d on B220^+^ splenocytes from *mb1-cre* and DKO mice. Results representative of one of four pairs of mice analyzed. **(C–E)** Nuclear factor-κB (NF-κB) in WT (*ric8*^fl/fl^) and *ric8*^fl/fl^*mb1-cre* splenic transitional and follicular B cells. Fixed purified B-cells from *mb1-cre* and *ric8*^fl/fl^*mb1-cre* mice were immunostained with a p65 antibody and analyzed using Image Stream. Shown are the gating strategy **(C)** and representative images **(D)** of p65 NF-κB and p65 NF-κB^−^ B cells. Shown **(E)** are the % of T1/2 and FO cells harboring nuclear p65 NF-κB. A cell was considered with a nuclear translocation when at least 60% of total p65 NF-κB was in the nucleus. Results shown are mean ± SEM from four animals per group. **(F)** Representative flow cytometry results from WT (Gnai2^fl/fl^) and DKO splenic mouse B cells analyzed for their intracellular level of phosphorylated Bruton’s tyrosine kinase. Results from one of three pairs of mice analyzed.

To better understand the role of Gα_i_ proteins in MZ B cell development in mice, we used several different mouse lines. Mice with a B cell-specific loss of Gα_i2_ were generated by crossing *Gnai2*^fl/fl^ mice to *mb1-cre* mice. Double knockout (DKO) mice, which lack both Gα_i2_ and Gα_i3_ in their B cells were developed by crossing *Gnai3^−/−^* mice to the *Gnai2*^fl/fl^*mb1-cre* mice (24). We also crossed the *mb1-cre* mice to the *ric8*^fl/fl^ mice. Ric-8A functions as a chaperone and guanine nucleotide exchange factor for a subset of G protein α subunits including Gα_i_. The B cell specific loss of Ric-8A causes a marked reduction in B cell expression of Gα_i2_ and Gα_i3_ (30). Finally, we deleted *Gnai2* in hematopoietic cells using *vav1-cre*, which results in the loss of *Gnai2* at all stages of B cell development (31). To compare MZ development in these various mouse strains, we prepared spleen cells from them and analyzed cells in the B220 gate for their expression of CD21 and CD23. MZ B cells are CD21^high^ and CD23^low^, while FO B cells are CD23^high^ and CD21^int^ (Figure 1A). As we have previously noted MZ B cell development is largely intact in the *Gnai3^−/−^* mice, markedly reduced in the mice with a B cell specific deletion of *Gnai2* and in *Gnai2*^fl/fl^*vav1-cre* mice, and severely reduced in the DKO and *ric8*^fl/fl^*mb1-cre* mice (Figure 1A). We confirmed the loss of MZ B cells in the DKO mice by alternatively immunostaining with CD23 and CD1d. As expected we observed a sharp reduction in the B220^+^CD23^low^ and CD1d^high^ cells in the DKO mice (Figure 1B). Besides the reduced % of MZ B cells, there is also an overall loss of B cells in the spleen as has been previously documented (*Gnai2*^fl/fl^*mb1-cre, Gnai3^−/−^, Gnai2*^fl/fl^*vav1-cre, ric8*^fl/fl^*mb-1cre*, and DKO mice have approximately 92, 94, 88, 72, and 50%, respectively, of the number of splenic B cells in control mice) (24, 30, 31). A detailed picture of the % of cells among the different splenic B cell subsets in the various Gα_i_-deficient mice is shown in Table 1.

**Table 1:** Splenic B cell subsets in Gα_i_-deficient mice.

	*Gnai2*^fl/fl^ (*n* = 9)	*Gnai3^**−**/**−**^*(*n* = 3)	*Gnai2^fl/fl^mb1-cre*(*n* = 8)	Double knockout (*n* = 7)
Pre-B	1.253 ± 0.093	1.412 ± 0.425	1.793 ± 0.141	5.255 ± 0.412
Transitional B cell type 0	4.619 ± 0.588	4.839 ± 1.295	2.805 ± 0.453	6.697 ± 0.903
T1	10.098 ± 1.181	5.920 ± 1.250	8.248 ± 0.753	5.054 ± 1.411
Transitional B cell type 2-FO	9.764 ± 0.578	9.658 ± 0.362	7.101 ± 0.560	8.029 ± 0.330
FO	54.727 ± 2.841	57.880 ± 1.271	68.734 ± 1.928	70.483 ± 1.287
Marginal zone precursor	8.370 ± 1.051	8.529 ± 0.296	5.879 ± 0.486	4.340 ± 1.199
Marginal zone	10.348 ± 1.567	5.772 ± 0.869	2.340 ± 0.269	0.327 ± 0.132

*Results of immunophenotyping spleen B cells from Gα_i_ and wild-type mice*.

### Gα_i_-Deficient Transitional B Cells Have Similar Levels of Nuclear p65 and Phosphorylated Bruton’s Tyrosine Kinase (pBTK)

Since the strength of BCR signaling in transitional B cells is linked to the MZ versus FO B cell fate decision (8, 32), we checked whether we could find evidence of enhanced BCR signaling in Gα_i_-deficient transitional B cells, which would help explain their lack of MZ B cells. First, we used IS cytometry to examine the nuclear localization of p65 NF-κB in transitional and FO B cells from WT mice and from mice whose B cells largely lacked Gα_i2/3_ (*ric8*^fl/fl^*mb1-cre*). We found that approximately 38, 47, and 45% of the WT transitional, FO, and MZ B cells, respectively, had nuclear p65. However, these results did not differ from those obtained with the B cells fractions deficient in Gα_i_ proteins (Figures 1C–E). Next, we assessed the status of BTK by examining the levels of BTK phosphorylated on Y223 in transitional and FO B cells. Located in the BTK SH3 domain, this is an autophosphorylation site, whose phosphorylation occurs following Src kinase Y511 transphosphorylation and that can be induced by IgM crosslinking (33). Normalized to the WT T0 cells, the WT T1 and T2, and FO cells had a 19, 53, and 48% increase in pBTK, respectively (Figure 1F). Compared to their respective wild-type fractions, the DKO T0 and T1 fractions had an approximately 12% decrease in pBTK, the DKO T2 cells had a similar level, while the DKO FO B cells had a 35% increase. Overall, these results do not support a role for enhanced BCR signaling in the Gα_i_-deficient B cell transitional B cells as a contributing factor to the lack of MZ B cells in these mice.

### Decreased Notch Signaling in B Cells Lacking Gα_i_ Expression

**Figure 2:**
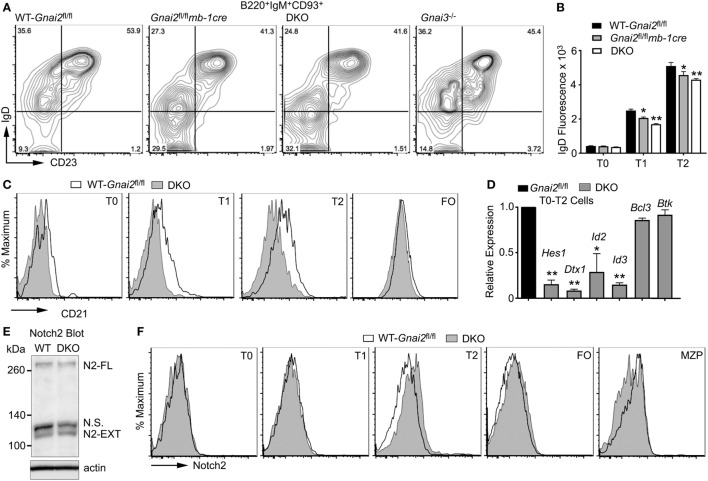
Impaired Notch signaling, but unaltered Notch2 receptor expression in Gα_i_-deficient B cells. **(A)** Flow cytometry of splenic B cells from the indicated mice gated on B220^+^IgM^+^CD93^+^ for their expression of IgD versus CD23 to distinguish transitional B cell type 0 (T0), transitional B cell type 1 (T1), and transitional B cell type 2 (T2) cells. Results representative of four mice of each genotype. **(B)** Flow cytometry to determine IgD expression on WT, Gα_i2_, and Gα_i2/3_-deficient T0, T1, and T2 transitional cells. Splenic B cells prepared from WT, *Gnai2*^fl/fl^*mb1-cre*, and double knockout (DKO) mice were analyzed for B220, CD93, IgM, IgD, and CD23 expression. Results from the analysis of three mice of each genotype. Mean fluorescence data are shown as the mean ± SEM of the relative expression of each gene over control cells for the indicated subset (**p < 0.005, *p < 0.05). **(C)** Flow cytometry showing CD21 expression on WT (*Gnai2*^fl/fl^) and DKO transitional, and FO B cells. Results are representative of six or more mice of each genotype. **(D)** RT-PCR analysis of splenic B cells from WT and Gα_i_-deficient mice. Expression levels of the indicated gene were assessed in mRNA extracted from B220^+^IgM^+^CD93^+^ cells sorted from control (*Gnai2*^fl/fl^) and DKO mice. The results are from two controls versus two DKO. Experiment repeated twice with a similar result. Data shown are the mean ± SEM of the relative expression of each gene over control cells for the indicated subset (**p < 0.005, *p < 0.05). **(E)** Immunoblot of Notch2 in WT (*Gnai2*^fl/fl^) or DKO B cell lysates. Experiment performed twice with a similar result. N.S. delineates a non-specific band. **(F)** Flow cytometry analysis of Notch2 receptor expression in spleen cell subsets from WT (*Gnai2*^fl/fl^) and DKO mice. Representative results from one of three experiments.

Upon entering the splenic white pulp, T0 B cells acquire surface IgD and develop to T1 B cells (10). However, Gα_i_-deficient B cells have difficulty entering the white pulp (24). Consistent with this defect, the Gα_i_-deficient B cells exhibit a partial T0 to T1 transition block. This can be visualized by flow cytometry by gating on the B220, IgM, and CD93-positive cells and plotting IgD versus CD23. Such profiles show an increased ratio between the T0 and T1 cells from the *Gnai2*^fl/fl^*mb1-cre, ric8*^fl/fl^*mb1-cre*, and DKO mice as compared to the same ratio using cells from the WT and *Gnai3^−/−^* mice (Figure 2A). Furthermore, the Gα_i_-deficient T1 and T2 transitional B cells expressed a lower level of surface IgD than did the WT transitional B cells (Figure 2B). A failure to properly enter the white pulp could limit Gα_i_-deficient B cells from contacting the Notch ligands present on CCL19-expressing follicular fibroblasts (19). To assess whether the Gα_i_-deficient B cells had received inadequate Notch signals, we first checked CD21 expression on transitional and FO B cells from WT and DKO mice. A known Notch2 target gene, the CD21 gene has binding sites for Rbp-J, a DNA-binding protein to which the NICD binds (34). Consistent with an early impairment in Notch signaling, the DKO transitional B cells had reduced levels of CD21 expression compared to control cells (Figure 2C). Next, we used RT-PCR to check the expression of several other Notch target genes including hairy and enhancer of split-1 (*Hes1*), Deltex1 (*Dtx1*), and inhibitor of DNA binding 2 (*Id2*). Hes1 is a basic helix loop helix transcription factor activated by Notch signaling, while Dtx1 is a ubiquitin ligase that regulates Notch signaling (35). Id2 and Id3 inhibit the DNA-binding activity of E proteins, transcription factors important for B cell development and differentiation (36). The control transitional B cells (T0–T2) expressed higher mRNA levels for *Dtx1, Id2, Id3*, and *Hes1* than did the DKO cells, while *Btk* and *Bcl3* mRNA levels were similar (Figure 2D). These data indicate that the Gα_i_-deficient transitional B cells had received inadequate *in vivo* Notch2 signaling. Either the Gα_i_-deficient transition B cells had not encountered Notch ligands, or if they had, they had failed to activate Notch signaling.

### Gα_i_-Deficient B Cells Have Adequate Notch2 Expression, but They Differentiate Poorly to MZ B Cells, Even in the Presence of Notch2 Ligands

To determine whether the Gα_i_-deficient B cells express normal levels of Notch2, we first examined B cell lysates by Notch2 immunoblotting, which revealed no differences in the level of unprocessed Notch2 or the N2-Ext domain (Figure 2E). To assess Notch2 surface expression, we used flow cytometry. We found similar levels of Notch2 expression on WT and DKO bone marrow progenitors (Figures 2A–E) and recirculating B cells (Figure 2F) (data not shown). Next, we checked various splenic B cell populations (Figure 2F). WT and DKO T0, T1, and FO splenic B cells also expressed similar levels. We did note a slight enhancement in Notch2 expression on the DKO T2 cells, and a reduced level on the DKO MZ precursor (MZP) cells. Thus, the reduction in Notch2 target genes noted in the Gα_i_-deficient B cells cannot be explained by inadequate levels of Notch2 expression at the plasma membrane.

**Figure 3:**
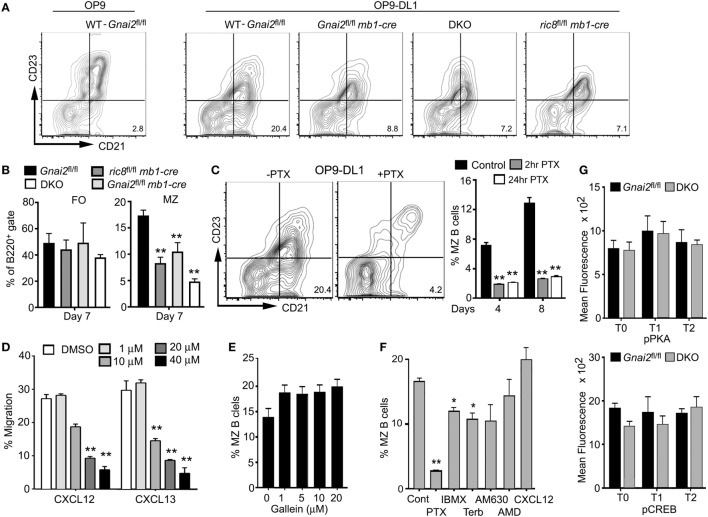
Even direct contact with Notch ligand expressing cells cannot rescue marginal zone (MZ) B cell development when Gα_i_ signaling is impaired. **(A)** Flow cytometry patterns of WT and Gα_i_-deficient bone marrow-derived B cells plated on OP9 or OP9-Delta-like 1 (DL1) cells for 7 days in the presence of BAFF. Prior to their transfer to the stromal cell, WT and Gα_i_-deficient bone marrow cells were cultured with IL-7 for 4 days. The % of MZ B cells is indicated in the lower right corner of each flow pattern. **(B)** Quantification of WT and Gα_i_-deficient FO and MZ B cell development using OP9-DL1 system. Results from analyzing cells prepared from at least four mice of each genotype. Data are mean ± SEM (**p < 0.005). **(C)** MZ B cell development in OP9-DL1 system using WT cells treated with pertussis toxin (PTX), or not. PTX was added to the bone marrow cell culture for the terminal 2 or 24 h. After washing the cells were cultured on OP-9DL1 cells for 4 or 8 days. Shown are representative flow patterns and overall results from three experiments. **(D)** The Gβγ inhibitor Gallein reduced B cell chemotaxis to chemokines. Splenic B cells were exposed to the indicated concentration of Gallein for 1 h prior to adding the cells to the upper chemotaxis chamber. An equal concentration of Gallein was present in the upper and lower chambers. The indicated concentration of CXCL12 was added to the lower chamber. Results are mean of three determinations for each concentration. Representative of one of three experiments performed. **(E)** Gallein does not inhibit the recovery of MZ B cells in OP9-DL 1 cultures. WT B cells were treated with the indicated concentration of Gallein, or not. The same concentration of Gallein was added to the culture every other day. Results from one experiment performed in duplicate. The experiment was repeated three times with comparable results. Data are mean ± SEM. **(F)** MZ B cell development in OP9-DL1 cultures is reduced by raising intracellular cyclic adenosine monophosphate (cAMP) levels. Bone marrow-derived B cell progenitors were plated on OP9-DL1 cells in the presence of IBMX (1 µm), terbutaline (10 nM), AM630 (1 µm), AMD3100 (1 µg/ml), or CXCL12 (1 mg/ml). The same concentration of each of the compounds was added on days 2, 4, and 6 except for PTX, which was only used to pretreat the cells prior to the OP9-DL1 culture. Results from 1 experiment performed in duplicate. The experiment was repeated three times with comparable results. Data are mean ± SEM (**p < 0.005, *p < 0.05). **(G)** No evidence of abnormal cAMP signaling *in vivo* in Gα_i_-deficient transitional B cells. Intracellular FACS to assess pCREB and pPKA levels in WT (*Gnai2*^fl/fl^) and double knockout (DKO) transitional B cells. Results from comparison of three WT and four DKO mice. Data are mean ± SEM.

To test whether we could restore MZ B cell development by ensuring access to Notch ligands, we used a previously describe *in vitro* assay of splenic B cell development (37). Non-adherent bone marrow cells that had been cultured for 4 days with Il-7 (>95% B220^+^CD19^+^CD93^+^) were plated on a stromal cell line that expresses a Notch ligand (OP9-DL1) or not (OP9), in the presence of BAFF. Phenotypic T0, T1, and T2 cells were evident 2 days later, and phenotypic FO and MZ shortly thereafter (data not shown, see below). By 7 days, approximately 15–25% of the recoverable cells had a MZ B cell phenotype. As expected, few if any MZ B cells appeared in the OP9 cultures. By contrast, directly plating the Gα_i_-deficient B cell progenitors on the OP9-DL1 cells did not rescue normal MZ B cell development (Figures 3A,B). These data argue that the Gα_i_-deficient transitional B cells are less sensitive to Notch ligands.

### Gα_i_ Nucleotide Exchange, but Not Gβγ Signaling, Is Needed for MZ B Cell Development in OP9-DL1 Cultures

To provide further evidence that Gα_i_ signaling is needed for MZ B cell development we made use of PTX, which ADP ribosylates Gα_i_ proteins preventing receptor triggered Gα_i_ nucleotide exchange. This inhibits Gβγ subunit release from the heterotrimeric G-protein, thereby blocking the activation of downstream effectors of Gα_i_-GTP and Gβγ (38). When we treated bone marrow B cell progenitors with PTX, prior to plating them on OP9-DL1 cells, we found a sharp reduction in the appearance of phenotypic MZ B cells. Initially, we added PTX during the final 24 h of the IL-7 culture, but subsequently, showed that a 2-h exposure had a similar inhibitory effect (Figure 3C). To assess the importance of Gβγ subunit release, we employed Gallein, a small molecule inhibitor of many Gβγ triggered signaling pathways including chemotaxis (39). We first established that Gallein inhibited B cell chemotactic responses (Figure 3D). It did, as a 20-µm concentration reduced B cell chemotaxis to either CXCL12 or CXCL13 by approximately two-thirds. However, repeatedly adding a similar concentration to the OP9-DL1 B cell cultures did not adversely affect MZ B cell development (Figure 3E). This result argues that a Gβγ effector function unaffected by Gallein treatment, or that a Gα_i_-GTP effector helps mediates MZ B cell development. Since the loss of Gα_i_ can affect intracellular cyclic adenosine monophosphate (cAMP) levels by allowing the unopposed action of Gα_s_, we assessed the impact of raising intracellular cAMP levels in the OP9-DL1 cultures. IBMX increases cAMP by inactivating cAMP phosphodiesterases while the β-adrenergic receptor agonist terbutaline raises cAMP *via* Gα_s_-mediated activation of adenylyl cyclases. While both compounds reduced the appearance of MZ B cells, neither approached the efficacy of PTX (Figure 3F). We also tested the impact of limiting CXCR4 or CB2 signaling by adding specific antagonists, AMD3100 and AM630, respectively (40, 41). However, both compounds had a negligible impact on MZ B cell development in the OP9-DL1 system (Figure 3F). We did note that adding CXCL12 slightly raised the % of MZ B cells at the termination of the culture although the difference did not reach significance. One caveat in interpreting these experiments, not only the progenitor B cells, but also the OP9-DL1 cells were exposed to the different compounds. However, this is unlikely an issue in the PTX experiments, as we exposed the progenitor B cells to PTX prior to adding them to the OP9-DL1 cells. Finally, to assess whether the loss of Gα_i_ proteins altered cAMP signaling *in vivo*, we checked phosphorylated protein kinase A and phosphorylated CREB levels in the WT and DKO transitional B cells by flow cytometry (Figure 3G). We found no significant differences between WT and DKO transitional B cells in this regard. Thus, while elevated cAMP levels may adversely affect MZ B cell development they are unlikely to account for the lack of MZ B cells when B cells lack Gα_i_ proteins.

### Reduced ADAM10 Expression in Gα_i_-Deficient Transitional B Cells

**Figure 4:**
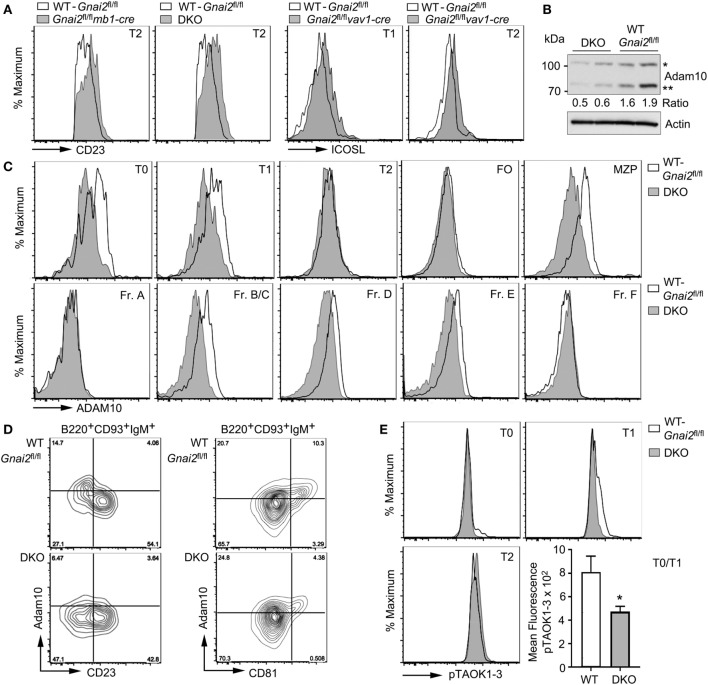
Reduced ADAM10 processing and expression in Gα_i_-deficient transitional B cells. **(A)** CD23 or ICOSL expression on Gα_i_-deficient B cells. Representative flow cytometry patterns of CD23 expression on transitional B cell type 2 (T2) cells from WT (*Gnai2*^fl/fl^) and either *Gnai2*^fl/fl^*mb1-cre* or double knockout (DKO) mice and ICOSL on transitional B cell type 1 (T1) and T2 cells from *Gnai2*^fl/fl^*vav1-cre* mice. **(B)** Immunoblot to detect ADAM10 expression in lysates from two WT (*Gnai2*^fl/fl^) and two DKO splenic B cells. Experiment repeated twice with equivalent results. The ratio between the unprocessed (*) and mature (**) ADAM10 is show. Actin immunoblot shown to verify equal loading. **(C)** Flow cytometry showing expression of ADAM10 in bone marrow and in splenic B cell fractions. Experiment was repeated a minimum of three times with a similar result. **(D)** ADAM10 expression on B220^+^CD93^+^IgM^+^ spleen cells as a function of CD23 or CD81 expression using cells from WT (*Gnai2*^fl/fl^) or DKO mice. Flow cytometry showing a plot of ADAM10 versus CD23 (left) or CD81 (right). The % of positive cells in each quadrant is indicated. **(E)** pTAOK expression in WT (*Gnai2*^fl/fl^) and DKO splenic B cells. Intracellular flow cytometry assessing phosphorylation of TAOK proteins in splenic B cells from WT and DKO mice. Quantification of pTAOK proteins in transitional B cell type 0 (T0) and T1 B cells as measured by flow cytometry from four WT and four DKO mice. Data are mean ± SEM (*p < 0.05).

Diminished Notch2 target gene expression following direct exposure to Notch2 ligands suggests defective Notch2 processing in the Gα_i_-deficient cells. We focused on ADAM10, whose activity releases the extracellular domain of Notch2 allowing γ-secretase to release the NICD2. The ADAM10 metallopeptidase functions not only to cleave the extracellular domain of Notch2 but also other surface membrane proteins including CD23 and ICOS ligand (42, 43). Consistent with a defect in ADAM10 sheddase activity, we found elevated levels of both membrane proteins on the Gα_i_-deficient T2 cells compared to WT cells (Figure 4A). Immunoblotting ADAM10 in cell lysates from WT and DKO B cells revealed that the DKO B cells had reduced levels of ADAM10, and impaired ADAM10 processing (Figure 4B). Maturation of ADAM10 requires removal of the prodomain by either Furin or PC7. The prodomain functions as a molecular chaperone important for the localization of the catalytic domain to the cell membrane (44). The DKO B cell lysates had 50% less processed ADAM10 compared to the WT B cell lysates. To assess ADAM10 surface expression, we used flow cytometry comparing both bone marrow and splenic B cell subsets. This analysis showed a reduction in ADAM10 expression on the DKO bone marrow Fr. B/C, Fr. D., and Fr. E cells and on DKO splenic T0 and T1 cells, and MZPs (Figure 4C). Of note, the Gα_i_-deficient T2 cells had elevated CD23 and ICOS ligand levels despite having ADAM10 levels equivalent to the WT T2 cells. This may reflect the reduced ADAM10 levels on the Gα_i_-deficient T1 cells or reduced ADAM10 activity despite a similar expression levels.

In the study of *Taok3^−/−^* mice (12), the reduction of ADAM10 expression on *Taok3^−/−^* T0 and T1 cells was shown by gating on CD19^+^CD93^+^IgM^+^ spleen cells, plotting ADAM10 expression versus CD23, and focusing on the ADAM10^+^CD23^−^ cells. A similar strategy shows the reduction in ADAM10 expression on the DKO cells as compared to the WT cells (Figure 4D). Because certain tetraspanins (TSPAN) can interact with ADAM10 and modify ADAM10 activity (45), we gated on CD19^+^CD93^+^IgM^+^ cells and analyzed ADAM10 versus CD81 (TSPAN28), CD63 (TSPAN30), CD53 (TSPAN25), or CD9 (TSPAN29). Of the different TSPANs, only CD81 proved useful as we found fewer ADAM10^+^ cells in the CD81^+^ subset of Gα_i_-deficient transitional B cells when compared to wild-type cells (Figure 4D). Finally, we checked the status of pTAOK1-3 by flow cytometry using a monoclonal antibody that recognizes phosphorylated S181 in TAOK1 and 2, and S177 in TAOK3 (Figure 4E). We found a small reduction in the amount of phosphorylated TAOK1-3 in the DKO T0 and T1 cells. Thus, the poor ADAM10 expression on T0 and T1 cells in the spleen helps to explain the defective MZ B cell development in mice whose B cells lack Gα_i2_ or Gα_i2_ and Gα_i3_.

### Both CXCR4 Engagement and Anti-IgM Crosslinking Increase TAOK, BTK, and CREB Protein Phosphorylation in Transitional T0 and T1 Cells

**Figure 5:**
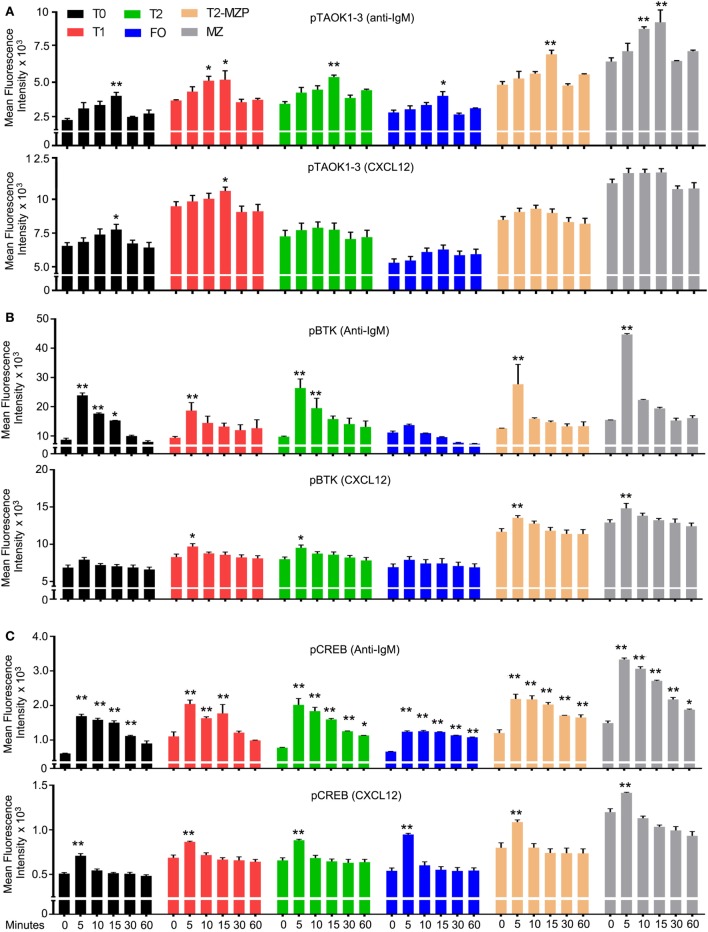
Both CXCL12 and anti-IgM raise the level of pTAOK, phosphorylated Bruton’s tyrosine kinase (pBTK), and pCREB in transitional B cells. **(A–C)** Intracellular FACS results from WT splenic B cells stimulated with either CXCL12 (1 µg/ml) or anti-IgM (10 µg/ml) for the indicated durations. Prior to addition of the stimulant, the B cells were incubated in media for 30 min. Following fixation and permeabilization, the cells were immunostained for expression of pTAOK **(A)**, pBTK **(B)**, or pCREB **(C)**. The results are from the analysis of splenic B cells from four mice (two versus two, performed twice). Data are mean ± SEM (*p < 0.05, **p < 0.005).

Next, we compared the effect of CXCL12 and anti-IgM on the phosphorylation status of TAOK proteins in various B cell subsets with a focus on transitional B cells. To do so, we exposed wild-type B cells to either anti-IgM or to CXCL12 and used flow cytometry to monitor pTAOK proteins in the different splenic B cell subsets (Figure 5A). We found that a 15-min exposure to either stimulus increased pTAOK1-3 in T0 and T1 cells, although anti-IgM proved more potent. Anti-IgM stimulation also raised the pTAOK1-3 levels in T2, MZP, and MZ B cells while CXCL12 did not. For comparison, we checked the same stimuli on the levels of pBTK and pCREB. Mice lacking BTK have a defect in the T2 to FO B cell transition, but do not have defective MZB cell development (46). We found that anti-IgM treatment triggered increases in pBTK levels in all B cell populations apart from FO B cells, while CXCL12 increased pBTK levels in all the B cell populations except for T0 and FO B cells. In each instance, CXCL12 triggered a lower, and less sustained response than did BCR crosslinking (Figure 5B). In non-hematopoietic cells, CREB phosphorylation has been linked to ADAM10 processing (47). We found that anti-IgM and CXCL12 increased pCREB levels in all the splenic B cell subsets although again BCR crosslinking led to higher, more sustained responses (Figure 5C). These results indicate that chemoattractant receptor and BCR signaling activate a partially overlapping set of signaling intermediates in transitional B cells.

### CXCL12 or BCR Crosslinking Raises ADAM10 Levels on WT T0/T1 B Cells, but Not on Gα_i_-Deficient T0/T1 B Cells

**Figure 6:**
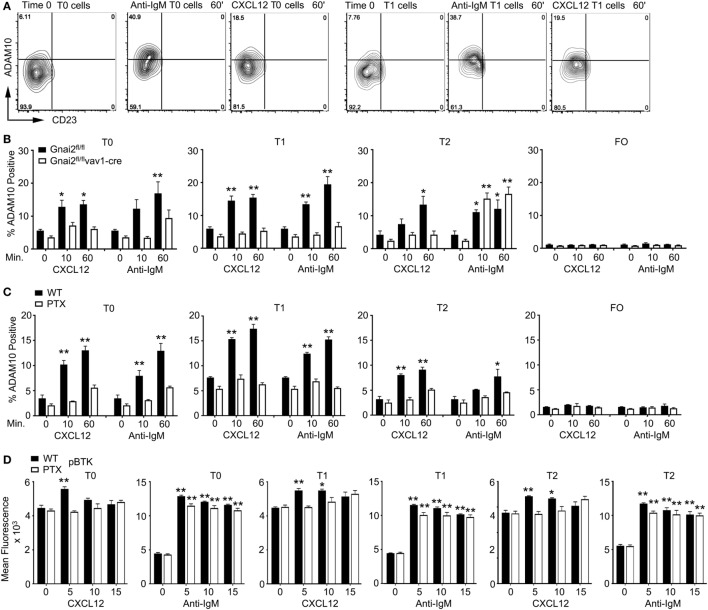
Anti-IgM or CXCL12 induces ADAM10 expression on transitional B cell type 0 (T0) and transitional B cell type 1 (T1) B cells. **(A)** Flow cytometry results examining CD23 versus ADAM10 on T0 and T1 B cells stimulated with anti-IgM or CXCL12. Purified spleen cells equilibrated for 30 min prior to stimulation with 10 µg/ml of anti-IgM F(ab′)_2_ or CXCL12 (1 µg/ml) for 1 h prior to analysis. The cells were gated on B220^+^CD193^+^IgM^+^CD23^−^ cell expressing IgD, or not, and the data displayed as ADAM10 versus CD23. **(B)** Flow cytometry results examining ADAM10 expression on transitional B cells from WT and *Gnai2*^fl/fl^*vav1-cre* mice. Purified spleen cells from the indicated mice equilibrated for 30 min prior to stimulation with 10 µg/ml of Anti-IgM F(ab′)_2_ or Cxcl12 (1 µg/ml) for 1 h prior to analysis. Data shown as % ADAM10 positive on T0, T1, transitional B cell type 2 (T2), or FO B cells. The results are from the analysis of splenic B cells from four *Gnai2*^fl/fl^ mice and four *Gnai2*^fl/fl^*vav1-cre* mice. Data are mean ± SEM (*p < 0.05, **p < 0.005) (comparison between basal and stimulated). **(C)** Flow cytometry results examining ADAM10 expression on transitional B cells. Purified spleen B cells were equilibrated for 2 h in media with pertussis toxin (PTX) (200 ng/ml), or not, prior to stimulation with 10 µg/ml of Anti-IgM F(ab′)_2_ or CXCL12 (1 µg/ml) for 1 h before analysis. Data shown as % ADAM10 positive on T0, T1, T2, or FO B cells. The results are from the analysis of splenic B cells from three WT mice, which were treated with PTX or not. Data are mean ± SEM (*p < 0.05, **p < 0.005) (comparison basal versus stimulated). **(D)** Intracellular flow cytometry to assess phosphorylated Bruton’s tyrosine kinase (pBTK) levels in transitional B treated with PTX or not. The results are from the analysis of splenic B cells from three WT mice, which were treated with PTX or not. Data are mean ± SEM (*p < 0.05, **p < 0.005) (comparison between basal and stimulated cells).

In the previous study of *Taok3^−/−^* mice, the analysis of wild-type, transitional B cells following BCR crosslinking ADAM10 translocated from the cytosol to the plasma membrane as assessed by immunocytochemistry (12). We performed a similar analysis although we followed ADAM10 membrane expression on transitional B cells by flow cytometry after exposure to anti-IgM or CXCL12. We found that both stimuli triggered increased ADAM10 expression on a subset of T0 and T1 B cells, while ADAM10 levels rose variably on T2 B cells, and not at all on FO B cells in response to the same stimuli. Shown are flow cytometry patterns from an experiment where anti-IgM induced a particularly strong increase in ADAM10 expression (Figure 6A, and data not shown). Next, we compared the response of WT and *Gnai2*^fl/fl^*vav1-cre* B cells to CXCL12 or anti-IgM. As expected, the Gα_i_-deficient B cells failed to mobilize ADAM10 to the cell surface following stimulation with CXCL12; however, surprisingly anti-IgM treatment also failed to raise ADAM10 expression on the Gα_i_-deficient T0/T1 B cells. By contrast, anti-IgM did increase ADAM10 expression on T2 B cells from the same Gα_i_-deficient mice (Figure 6B). To confirm these results, we assessed whether blocking Gα_i_ signaling in WT transitional B cells would also inhibit anti-IgM-induced upregulation of ADAM10, which it did (Figure 6C). In contrast to the *Gnai2*^fl/fl^*vav1-cre* T2 cells, the PTX-treated WT T2 did not respond to antigen receptor crosslinking. As expected, PTX treatment reduced the CXCL12-induced increases in pBTK levels in the T0, T1, and T2 cells (Figure 6D). In contrast to the sharp reduction in ADAM10 expression following exposure of the transitional B cells to anti-IgM, pBTK levels were minimally affected by the PTX treatment (Figure 6D). Thus, the loss of Gα_i_ proteins or Gα_i_ signaling not only impairs the upregulation of ADAM10 on T0/T1 B cells in response to CXCL12 but also following BCR crosslinking.

### The Increase of ADAM10 Levels in OP9-DL1 Cultures Depends Upon Gα_i_ Signaling

**Figure 7:**
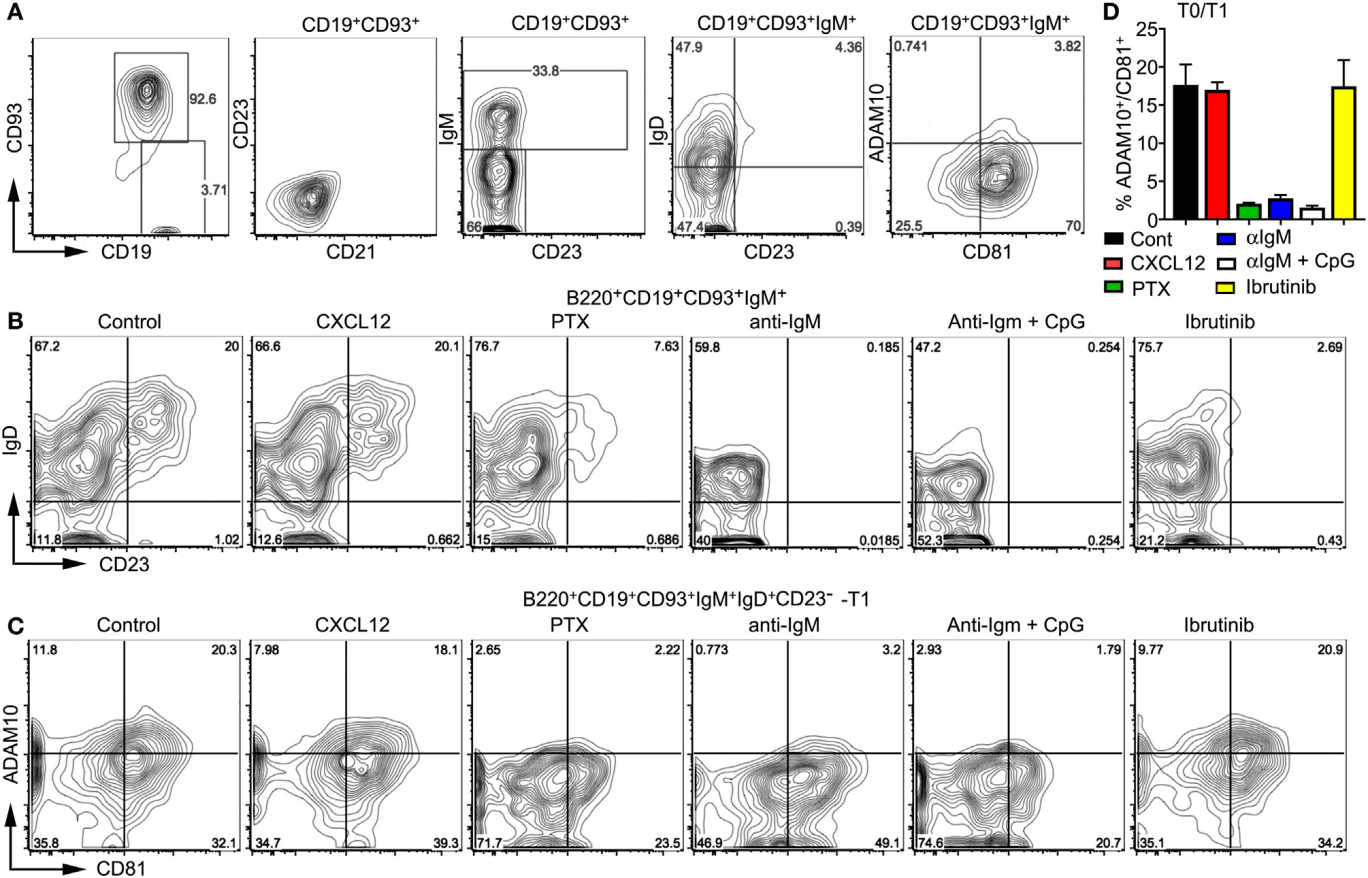
Gi signaling raises ADAM10 expression on transitional B cells. **(A)** Characterization of the bone marrow-derived progenitor B cells prior to their addition to OP9-DL1 cultures. Mouse bone marrow cells were cultured with IL-7 for 4 days after which the non-adherent cells were removed. The collected cells were characterized by flow cytometry as indicated. **(B,C)** Flow cytometry results of OP9-Delta-like 1 (DL1) cultures of bone marrow-derived B cells treated with pertussis toxin (PTX), CXCL12, anti-IgM, anti-IgM + CpGs, or Ibrutinib. Before transferring on to OP9-DL1 cells, some developing B cells were treated with PTX (200 ng/ml) for 2 h. On day 0 and day 1 after transfer to the OP9-DL1 cells, CXCL12 (200 ng/ml), anti-IgM (10 µg/ml), CpGs (1 µg/ml), or Ibrutinib (30 nM) were added. On day 2, the non-adherent cells were immunostained and analyzed by flow cytometry to assess IgD versus CD23 expression **(B)** or ADAM10 versus CD81 **(C)** on the cells gated as indicated. Comparable results were observed in two other experiments. **(D)** Chart showing % of ADAM10^+^CD81^+^ cells in OP9-DL1 cultures of WT bone marrow cells treated as in panel **(B)**. Analysis of transitional B cell type 0 (T0) and T1 cells 2 days after plating on OP9-DL1 cells. Data are from two independent experiments and are the mean ± SEM (*p < 0.05, **p < 0.005).

To further test the impact of BCR and Gα_i_ signaling on developing wild-type transitional B cells, we used bone marrow-derived B cell progenitors and the OP9-DL1 system. Following culture with IL-7 for 4 days, the bone marrow cells were 95% CD19^+^B220^+^CD93^+^. These cells lacked CD21 and CD23 expression, one-third expressed surface IgM, and of those one-half expressed IgD. Approximately 3% of the CD19^+^CD93^+^IgM^+^ cells expressed ADAM10 (Figure 7A). These cells were plated on OP9-DL1 cells in the presence of BAFF. Prior to plating we treated some cells with PTX for 2 h or not. To others cultures, we added CXCL12, anti-IgM, anti-IgM plus CpGs, or Ibrutinib, which inhibits Bruton’s tyrosine kinase (48). We analyzed the cells 2 days later. First, we assessed the relative percentage of T0, T1, and T2 cells finding that PTX, anti-IgM, anti-IgM plus CpGs, and Ibrutinib to varying degrees inhibited both the T0–T1 and T1–T2 transition. By contrast, the addition of CXCL12 had no effect (Figure 7B). Next, we analyzed the impact of the same stimuli on ADAM10 expression on developing T0 and T1 cells. We found that PTX, anti-IgM, and anti-IgM plus CpGs caused a reduction in the % of CD81^+^ADAM10^+^ cells, while CXCL12 and Ibrutinib had no effect (Figures 7C,D). These results indicate that Gα_i_ signaling supports ADAM10 expression on T0 and T1 B cells, providing a mechanism by which either constitutive Gα_i_ signaling or the engagement with GPCR ligands can promote MZ B cell development. By contrast, the persistent exposure of progenitor and transitional B cells to surface Ig crosslinking interferes with T0–T1 and T1–T2 transitions and blocks ADAM10 membrane expression.

**Figure 8:**
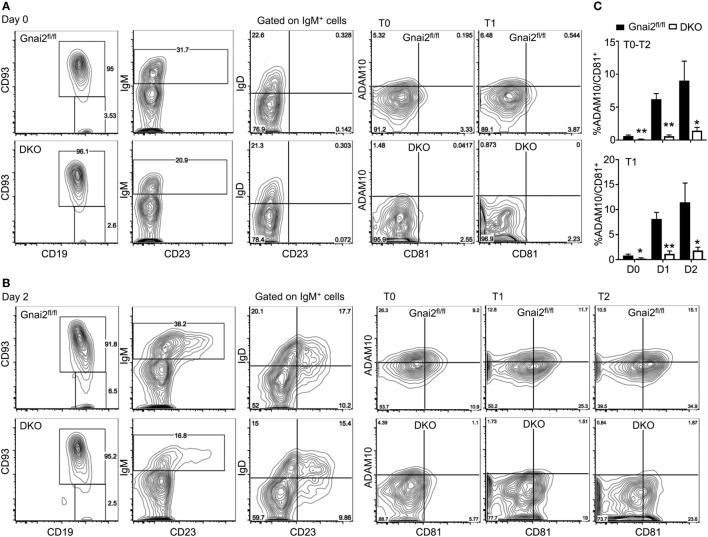
Developing Gα_i_-deficient transitional B cells in OP9-Delta-like 1 (DL1) cultures poorly upregulate ADAM10 expression. **(A)** Representative flow cytometry to analyze IL-7 cultured bone marrow cells. Bone marrow cells from *Gnai2*^fl/fl^ or double knockout (DKO) mice cultured with IL-7 for 4 days. Following removal of the non-adherent the cells, the remaining cells were immunostained for CD19, CD93, IgM, IgD, CD23, ADAM10, and CD81. Shown are CD19 versus CD93; IgM versus CD23 gated on CD19^+^CD93^+^; IgD versus CD23 gated on CD19^+^CD93^+^; CD81 versus ADAM10 gated on CD19^+^CD93^+^IgM^+^IgD^−^ [transitional B cell type 0 (T0)]; and CD81 versus ADAM10 gated on CD19^+^CD93^+^IgM^+^IgD^+^CD23^−^ [transitional B cell type 1 (T1)]. **(B)** Representative flow cytometry results to analyze B cell development and ADAM10 expression 2 days after plating on OP9-DL1 cells. Bone marrow cells from panel **(A)** further cultured on OP9-1 cell in the presence of BAFF. The developing B cells from the and DKO mice were characterized by flow cytometry as in panel **(A)**, but in addition CD81 versus ADAM10 expression is shown on CD19^+^CD93^+^IgM^+^IgD^+^CD23^+^ [transitional B cell type 2 (T2)] cells. **(C)** Charts of the % of ADAM10^+^CD81^+^ T0–T2 cells in OP9-DL1 cultures of WT and DKO bone marrow (above) or only the T1 cells (below). Results from the analysis of bone marrow prepared from three WT and four DKO mice. Data are mean ± SEM (*p < 0.05, **p < 0.005).

Finally, we confirmed the importance of Gα_i_ proteins in ADAM10 expression by checking the acquisition of ADAM10 by developing WT and Gα_i_-deficient B cells in the OP9-DL1 system. Following culture with IL-7 for 4 days, bone marrow cells from either control (*Gnai2*^fl/fl^ or *mb1-cre*) mice or from the DKO mice were plated on OP9-DL1 cells in the presence of BAFF. We analyzed the cells prior to plating on the OP9-DL1 cells, also one (data not shown), and 2 days after plating. Representative flow cytometry patterns demonstrating CD93 versus CD19 expression, IgM versus CD23, IgD versus CD23 on IgM^+^ gated cells, and ADAM10 versus CD81 on T0, T1, and T2 cells are shown (Figures 8A,B). Following culture with IL-7, nearly all the WT and DKO bone marrow cells expressed CD19 and CD93. However, a lower percentage of the DKO progenitors expressed surface IgM as compared to wild-type B cell progenitors. Among the WT and DKO IgM-positive cells, IgD levels appeared similar. We found a slight reduction in ADAM10 expression on the day 0 DKO T0 and T1 cells. After 1 day of culture on the OP9-DL1 cells, ADAM10 expression increased on the WT transitional B cells, while we noted little to no increase on the DKO T0–T2 B cells. After 2 days of culture, the percentage of ADAM10^+^ transitional B cells continued to increase among the WT cells, while only a minimal increase of ADAM10 expression occurred on the DKO transitional B cells. Of note, the DKO transitional B cells expressed lower amounts of CD81 compared to the control cells. The % of CD81^+^ADAM10^+^ cells among the T0–T2 and T1 transitional B cells at day 0, day 1, and day 2 are shown (Figure 8C). Thus, the inability of the Gα_i_-deficient progenitors to efficiently upregulate ADAM10 expression likely explains the lack of MZ B cell development in these cultures.

## Discussion

Whether or how Gα_i_ proteins promote MZ B cell development is unknown. In this study, we tested three different hypotheses: an inhibitory role for Gα_i_ proteins in BCR signaling in transitional B cells, an inability of Gα_i_-deficient transitional B cells to access Notch2 ligand expressing cells, and an intrinsic role for Gα_i_ proteins in Notch2 processing or signaling. This report supports the third hypothesis. Suggesting a defect in Notch2 processing or transcriptional activity both WT and Gα_i_-deficient transitional B cells expressed similar Notch2 levels, yet the latter exhibited impaired Notch2 signaling. The Gα_i_-deficient transitional B cells had low levels of CD21 and a reduction in *Hes1, Dtx1*, and *Id2* expression, known Notch2 target genes. Arguing that inadequate access to Notch2 ligands did not account for the MZ B cell development defect, plating Gα_i_-deficient B cell progenitors directly on a source of Notch ligands did not rescue MZ B cell development. Supporting a defect in Notch processing and/or translocation, the Gα_i_-deficient transitional B cells had reduced plasma membrane localized ADAM10 and either blocking Gα_i_ nucleotide exchange or the lack of Gα_i_ proteins inhibited progenitor B cells from upregulating ADAM10 expression. Together, these data indicate that Gα_i_ signaling helps supports ADAM10 expression and Notch 2 signaling in transitional B cell.

Notch processing and subsequent transcriptional activity depends upon its cleavage by membrane localized ADAM10. Besides lacking MZ B cells, ADAM10-deficient transitional B cells express low levels of Notch target gene (14). An ADAM10 processing defect partially accounts for the reduced membrane ADAM10 expression noted in the Gα_i_-deficient transitional B cells as immunoblots revealed a threefold to fourfold reduction in the mature form of ADAM10. Supporting this observation, anti-IgM did not trigger ADAM10 to translocate to the plasma membrane in the Gα_i_-deficient T0 and T1 B cells. Subject to multiple layers of regulation, ADAM10 activity requires proprotein convertases within the secretory pathway to cleave the ADAM10 zymogen. This releases the active enzyme and promotes its plasma membrane translocation. Removal of the prodomain depends upon a canonical site for a Furin-mediated cleavage located between the pro- and catalytic domains of ADAM10 (44). Previous reports have linked ADAM10 processing to GPCR signaling (49). Most of these studies focused on GPCR engagement triggering ADAM10-mediated EGF receptor transactivation rather than Notch2 activation. For example, treatment of non-transformed N15C6 cells with CXCL12 enhanced ADAM10 prodomain removal and triggered ADAM10-dependent amphiregulin ectodomain shedding (50). The enhanced ADAM10 activity depended upon Src kinase activity. Both free Gβγ subunits and GTP bound Gα_i_ have been implicated in Src kinase activation (51). Not only did the Gα_i_-deficient transitional B cells have reduced membrane ADAM10 expression but so did developing B cells in the bone marrow. Thus, the microenvironmental exposure to chemoattractants in the bone marrow and spleen likely supports the maturation of ADAM10 in developing B cells.

While a processing defect may explain the low ADAM10 membrane expression on the Gα_i_-deficient transitional B, Gα_i_ signaling also promotes the rapid translocation of ADAM10 to the plasma membrane. Only 5–10% of the wild-type transitional B cells (B220^+^CD93^+^IgM^+^CD23^−^) have elevated ADAM10 expression. It is this population that contained MZ, but not FO B cell precursors, and its loss in the *Taok3^−/−^* mice that accounted for the MZ B cell development defect (12). Like the *Taok3^−/−^* B cells, the Gα_i_-deficient B had a similar reduction in this population. Since ADAM10 processing occurred normally in the *Taok3^−/−^* transitional B cells, an ADAM10 translocation defect best explained their poor MZ B cell development. In addition, crosslinking surface Ig on wild-type B cells mobilized ADAM10 from intracellular stores to the plasma membrane as assessed by immunohistochemistry (12). Using flow cytometry, we confirmed that BCR crosslinking increased plasma membrane ADAM10 expression on transitional B cells. Although not as robust as surface IgM crosslinking, the exposure of transitional B cells to CXCL12 also led to increased ADAM10 surface expression. In line with these results, a brief exposure to either anti-Ig or CXCL12 increased the level of pTaok proteins in wild-type T0 and T1 cells.

However, bone marrow cell-derived B cell progenitors behaved differently when exposed to CXCL12 or anti-Ig. A 2-day culture on OP9-DL1 cells upregulated ADAM10 and CD23 expression. Perhaps because the OP9-DL1 cells secrete CXCL12 (52) and likely other GPCR ligands, the addition of exogenous CXCL12 to the OP9-DL1 cultures had no effect. However, pretreating the B cell progenitors with PTX strongly inhibited the induction of ADAM10, reduced CD81 expression, decreased the appearance of T2 B cells, and decreased MZ B cell development. Surprisingly, the addition of anti-IgM also inhibited CD23 upregulation and totally blocked the appearance of T2 cells. While a BTK inhibitor also prevented the appearance of T2 transitional cells, it did not affect ADAM10 or CD81 expression. Based on these studies, both antigen receptor crosslinking and G_i_ signaling can promote the membrane expression of ADAM10, a necessary step in splenic MZ B cell development. However, the timing and the context of the antigen receptor signal may be important in determining whether newly arrived bone marrow B cells arrest, or further differentiate toward either a FO or MZ B cell.

Signaling pathways triggered by Gα_i_ nucleotide exchange or downstream of BCR engagement cause the translocation of ADAM10 to cell membrane in transitional B cells. They may share or use divergent intermediates to trigger the translocation of ADAM10. Both BCR signaling and CXCR4 engagement enhanced the phosphorylation of Taok proteins, arguing that BCR and Gα_i_ signaling may converge on Taok3 activation, which either directly or indirectly controls the translocation of ADAM10. An intriguing target of Taok3 kinase activity is Mst1 (53). Taok3 functions in the Hippo pathway both in parallel and upstream of Mst1/2 to activate LATS1/2. In addition, Mst1 is also a known effector triggered by chemokine receptor engagement, whose activation depends upon Gα_i_ signaling. Interestingly, the *mst1^−/−^* mice also have a severe defect in MZ B cell development (54). It will be of interest to check whether the *mst1^−/−^* transitional B cells also have a reduction in ADAM10 membrane expression.

The eventual target of these signaling pathway must be ADAM10 or an ADAM10 associated protein. Intriguingly, a subgroup of tetraspanins (Tspan5, 10, 14, 15, 17, and 33) that have eight cysteines, referred to as the TspanC8 directly interact with ADAM10 (55). They affect the exit of ADAM10 from the endoplasmic endothelium, its translocation to the plasma membrane, and even its substrate specificity. Based on their mRNA expression (http://www.immgen.org/databrowser/index.html), the best candidate among the TspanC8 group to control ADAM10 expression in murine transitional B cells is Tspan14. Unfortunately, Tspan14 antibodies are not available for flow cytometry. However, since tetraspanin proteins often interact with other family members to form a tetraspanin web, we checked whether the expression of other family members might correlate with ADAM10 expression on transitional B cells. This led to the findings that the CD81^high^ transitional B cells also expressed membrane ADAM10 and that the Gα_i_-deficient transitional B cells, not only expressed less ADAM10, but also less CD81. In agreement with these results, a previous study showed that CD81 can regulate ADAM10-mediated cleavage of TNF-α and epidermal growth factor (56).

To our knowledge, GPCR or chemoattractant receptor signaling has not been linked to ADAM10 function and Notch signaling in B lymphocytes. Here, we showed that Gα_i_-deficient transitional B cells have decreased ADAM10 expression and reduced Notch2 target gene expression. Explaining the reduced ADAM10 expression on transitional B cells, we identified both an ADAM10 processing and a translocation defect. We suspect that these may be controlled by separate signaling pathways, likely downstream of different effectors. Reduced or absent Gα_i_ protein expression in developing B cells results in a prolonged absence of input from chemoattractant receptors, which likely supports both ADAM10 expression and processing in developing B cells. By contrast, PTX treatment acutely inhibits Gi signaling by prevents chemoattractant triggered Gα_i_ nucleotide exchange. Normal developing transitional B cells treated with PTX fail to mobilize ADAM10, while stimulation with CXCL12 rapidly mobilized ADAM10 to the cell membrane on a subset of T0/T1 cells. Both chemoattractant and BCR signaling triggered the translocation of ADAM10 from intercellular stores to the plasma membrane perhaps by targeting tetraspanins. Future studies will focus on unraveling the signaling pathways that control the expression, processing, and translocation of ADAM10 in transitional B cells.

## Ethics Statement

This study was carried out in accordance with the recommendations of the NIH Animal Research Advisory Committee (ARAC) guidelines, NIAID Animal Care and Use Committee. The protocol was approved by the NIAID Animal Care and Use Committee.

## Author Contributions

I-YH, CB, and JK designed and analyzed the experiments. CB, I-YH, and KH did the experimental work; CB and JK wrote and edited the manuscript.

## Conflict of Interest Statement

Dr. Boularan performed this work as part of a post-doctoral fellowship in the Laboratory of Immunoregulation, NIAID, NIH. The work was completed prior to the current affiliation of InvivoGen, therefore no conflict of interest exists. All other authors have no competing interests to disclose.

## Abbreviations

ADAM10, disintegrin and metalloproteinase domain-containing protein 10; BTKm, Bruton’s tyrosine kinase; DKO, *Gnai3^−/−^Gnai2*^fl/fl^*mb1-cre* mice; FO, follicular B cell; GEF, guanine exchange factor; GPCR, G protein-coupled receptor; NF-κB, nuclear factor-κB; PTX, pertussis toxin; Ric-8A, resistance to inhibitors of cholinesterase 8A; MZ, marginal zone; MZP, MZ precursor; T0, transitional B cell type 0; T1, transitional B cell type 1; T2, transitional B cell type 2; TSPAN, tetraspanin.
